# Application of catastrophe theory in comprehensive ecological security assessment of plastic greenhouse soil contaminated by phthalate esters

**DOI:** 10.1371/journal.pone.0205680

**Published:** 2018-10-31

**Authors:** Wei Zhou, Tingting Ma, Like Chen, Longhua Wu, Yongming Luo

**Affiliations:** 1 School of Civil Engineering and Architecture, Hubei University of Arts and Science, Xiangyang, China; 2 Institute of Hanjiang, Hubei University of Arts and Science, Xiangyang, China; 3 Key Laboratory of Soil Environment and Pollution Remediation, Institute of Soil Science, Chinese Academy of Sciences, Nanjing, China; 4 Shanghai Research Institute of Chemical Industry, Shanghai, China; 5 Key Laboratory of Coastal Zone Environmental Processes, Yantai Institute of Coastal Zone Research, Chinese Academy of Sciences, Yantai, China; Sun Yat-Sen University, CHINA

## Abstract

Large amount of phthalate esters (PAEs) used as plasticizers in polyvinyl chloride (PVC) products has caused ubiquitous contamination to the environment and potential ecology security risk all around the world, especially in places plastic films were indispensably utilized due to the widely proposing of facility agriculture in China. A case of PAEs contamination in four suburb areas of Nanjing was analyzed and discussed in this study. A new frame work has been put forward based on multi-criteria evaluation model and mathematical method of catastrophe theory, using farming work, laboratory determination and relevant environmental standards to measure the ecology security risk of PAEs in study areas. The factors were selected based on the availability of the data and the local conditions. The assessment model involves the contamination status of PAEs in soil and vegetables, the contamination effects of PAEs to human and soil organisms and the contamination source of PAEs from plastic films and other products in the four study facility agriculture areas. An evaluation system of the model was composed of thirteen mesosphere indicators and twenty-five underlying indicators including total PAEs concentration in soils, single PAE concentration in soils, total PAEs concentrations in roots, leafy, solanaceous and stem vegetables, PAE human risks, soil microbial counts, microorganism diversity indices, atmospheric deposition of PAEs, whether sewage wastewater irrigation, planting mode of the facility agriculture areas and climate condition of study areas. The modified evaluation system was used in the assessment of ecology security of the same place based on the data of 2012, and the results suggested that the ecology security indicators were reliable and were agree well with the practical situation of the study areas. The results could provide guidance for the application of health risk assessment of soil environment for the strong objectivity of catastrophe theory compared with other evaluation methods.

## Introduction

Agricultural production is the foundation of human society sustainable development, providing food crops, economic crops, vegetables and fruits, which could not only solve the problem of food and clothing for human survival and reproduction but also provide the necessary nutrients for human survival and health. As the development of the society and agriculture, with the continuous increasing of the population and the attendant urgent needs of more crops and vegetables in China, the mode of facility agriculture production has become a remarkably popular and dominant means of production. Essential to facility agriculture production, the employment of agricultural films has caused more trouble than the original “white pollution” problem in recent years on farmland in China. The main target pollutants of this study, phthalate esters or phthalic esters (PAEs), which have been utilized all over the world as the most common plasticizers, are the most critical component in films of facility agriculture and are easy to release into different environmental matrix and cause damage to different groups of organisms [1–4]. Because of the pressing need of increasing agricultural output and income of farmers, the production and inputs of agricultural films has been promoted at a hitherto unknown speed, which convinced the growing pollution trend of PAEs in farmland ecosystem. Particle precipitation after volatilization from and direct release of PAEs from plastic film are supposed to be the main sources in soils in polytunnel greenhouses [5]. During the management of the intensive vegetable cultivation, exponentially increased application of fertilizers and pesticide dose, plus the long time high temperature and high humidity of the internal environment of the greenhouses, has made the characteristics of the soil and the releasing of the PAEs in plastic films much easier. At the same time, the huge quantity of plastic film consumption has forced the farmers to choose films at low price and low quality for cost reduction. PAEs has been less of a concern than pesticides and heavy metals in the production of greenhouse vegetables [6], but after a half century of supposedly safe use, the impacts of PAEs on ecological systems and human health raised serious concerns [7,8]. In facility agriculture production areas, the ecology security risk caused by target PAEs pollutants should be taken into consideration and focused on because the intake of PAEs could be both carcinogenic and reproductive toxic [9].

Catastrophe theory originated as a branch of topology designed to deal with discontinuous dynamic systems governed by a potential function and can be used as a modeling approach to analyze complex nonlinear systems and has been applied in fields such as biology, physics, ecology and so on [10–12]. Date back to last century, catastrophe theory has been applied in a lake ecosystem to evaluate whether the introduction of more predatory fish could improve water conditions significantly [13]. A new framework has been set up using catastrophe theory, laboratory experiment, field work, and 3S (geographic information system, global positioning system, and remote sensing) to explore soil fertility self-development in the Zhuxi watershed of Changting County in the red soil hilly region of China [14]. Catastrophe theory also provided the conceptual framework for retrospective assessment of the impact of commercial grazing and soil water availability on the soil stability index (SSI), indicating that, the landscape became more susceptible to erosion events under multiple droughts and grazing [15]. A catastrophe model integrated multiple assessment indices of land eco-security according to the inherent contradictions and relative importance of indices without calculating weights were setup for assessing land ecological security in Shanghai, China [16]. A geospatial assessment framework, by integrating remote sensing, geographical information systems, landscape metrics, geo-statistics and catastrophe theory, was proposed and applied to characterize the spatial variations of agroecosystem health for a typical region in the eastern coastal agricultural plain, China [17]. However, in the assessment of soil ecology risk of organic pollutants, especially on PAEs, have been rarely seen up till now. It is necessary to combine practical data with the theoretical analysis results and to construct an integrative and comprehensive model. So, we developed a catastrophe model for the assessment of ecology security of the selected four study area contaminated by PAEs, selecting the environment and the living creatures as the most noteworthy factors based on the weight of data from 2011, to obtain a better understanding of the ecology security risk of target PAEs.

### Study area

As the capital of Jiangsu Province, a sub-provincial city, Nanjing is considered to be an important central city in eastern China, also the national key scientific research and education base and comprehensive transportation hub. Located in the lower reaches of the Yangtze River, the city has a population of 8.24 million in the dimensionality of about 659 700 hm^2^. The landform of Nanjing belongs to the hilly area among Nanjing, Zhenjiang and Yangzhou, and the region has a subtropical humid climate with four distinctive seasons and abundant rainfall.

Agriculture is the major industry of Nanjing city, and the total area of facility agriculture has reached over 17 860 hm^2^ until the year of 2008, which consists 7.4% of the total farmland area. Due to the large consumption of plastic films in facility agriculture production, the peasants are apt to choose plastic films that are cheaper and lower quality in order to save the cost of production. The selected four typical facility agriculture area in Nanjing, GL (118°41′41″E, 31°51′40″N), HS (118°59′57″E, 31°51′14″N), PLK (119°6′3″E, 31°34′40″N) and SS (118°58′55″E, 32°4′23″N), mainly use small arch shed and the quality of plastic films were relatively poor. Moreover, there has been no formal direction of relevant policies on the recycling of old plastic films so that the residual of phthalate esters (PAEs) coming from plastic films might cause the serious contamination to both the quality of vegetables produced in these areas and the environment, which entitled the processing of ecological security assessment of farmland soil contaminated by phthalate esters in the study area.

## Method

### Sampling and analytical method for soils and vegetable samples [6]

Soil and vegetable samples from 61 out of approximately 500 plastic film greenhouses for vegetable production in the four study suburban areas were selected in December 2011, considering the environmental status contour in terms of the distribution of agriculture and industry, hydrogeological conditions of nearby rivers, and features of greenhouses (age and species of vegetables). From the selected 61 greenhouses, a total of 305 surface (0∼15 cm) soil and vegetable paired samples (using quincunx sampling method in each greenhouse) were collected. The soil samples were collected by using a soil corer, while the plant samples were selected randomly for five fruit and compared after one quarter of each fruit was cut, mixed, and analyzed with three replicates. The fresh edible parts of each vegetable sample were collected and brought to the laboratory, washed with tap water, rinsed with distilled water, and wiped dry with paper tissue. Both vegetables and soils were then freeze dried in a Free Zone 2.5-Liter Freeze Dry System (Labconco Corp., Kansas City, MO). Ten grams of dried soils was ground and sieved (60 mesh), and 2 g of vegetable sample was homogenized for each replication in liquid nitrogen prior to storage at -20°C for subsequent analysis.

Glassware was washed by strictly following the procedure described by Ma et al [18] prior to analysis. Sample processing procedure was conducted following the description of Ma et al [18] for soils and Ma et al [19] for vegetables. 10 μL of internal standard (BB) was added before hexane (HPLC grade) was added to bring the final volume to 1 mL. Samples were transferred to brown sample bottles and stored at -20°C before further analysis. Analysis of individual PAEs in samples was performed exactly following the description of Ma et al [19] modified from USEPA method 8270C with an Agilent 7890GC-5975 MSD. Quality assurance and quality control results showed the high accuracy and sensitivity of this method and the reliability of the results [19]. For every16 samples, two whole procedure blanks, two soil matrix blanks, and one certified reference material (CRM) 136–100 were analyzed to ensure the analysis reliability.

The study of the selected samples in four areas was carried out on private lands, and we confirm that the owner of the land gave permission to conduct the study on these sites. We state clearly that no specific permissions were required for these locations/activities, because the farmers agreed with our behavior of sampling and investigation on the soils and vegetables. We confirm that the field studies did not involve endangered or protected species.

### Catastrophe theory

Catastrophe theory is a mathematical subject that was proposed by Rene Thom [20] aiming at rationally account for the phenomenon of discontinuous change in behaviors resulting from continuous change in different parameters in a given system [16]. In catastrophe theory, system function variables are divided into dependent state variable, which are the internal token variables of system, and control variables, which are the external influence factors while system is running [21]. Catastrophe theory is widely used, and one common application is the use of the catastrophe progression method derived from catastrophe theory to solve multiple criteria decision problems [22]. Based on catastrophe theory, the multi-criteria evaluation method draws on analytic hierarchy, utility function and fuzzy evaluation to obtain catastrophe fuzzy membership functions by normalized treatment of the bifurcation set [23], in which the dependency of state variables on control variables is determined by the catastrophe fuzzy membership functions, rather than weights assigned by the users and different control variables have different impacts on state variables in the multi-criteria evaluation method [24].

In the multi-criteria evaluation method, the system is divided into several subsystems with different evaluation indicators according to the inner mechanisms of the system being assessed. The initial data from the underlying layers are then normalized using catastrophe theory and fuzzy mathematics to give the optimal or cleanest data. To accomplish this, multidimensional catastrophe fuzzy membership functions assign values ranging from 0 to 1 to resolve the incomparability of various initial data induced by differences in the data span and dimensions. The total catastrophe fuzzy membership functions of the system are then determined by the normalized data [23]. When normalizing the initial data from the underlying layers, the cleanest data should be set to 1, after which the remaining data are converted to catastrophe fuzzy membership functions with values ranging from 0 to 1. After being broken down according to their impacts on the higher-level indicators, the evaluation indicators from different layers are prioritized.

There are four types of catastrophe fuzzy membership functions with varying numbers of indicators: the fold catastrophe model, which has one evaluation indicator (u_1_); the cusp catastrophe model, which has two evaluation indicators (u_1_ and u_2_); the swallowtail catastrophe, which has three evaluation indicators (u_1_, u_2_, and u_3_); and the butterfly catastrophe, which has four evaluation indicators (u_1_, u_2_, u_3_ and u_4_) listed in Table 1 [25].

**Table 1:** Description of catastrophe models.

Category	Dimension of control variables	Potential function	Bifurcation set	Normalization formula
**Fold model**	1	*V*(*x*) = *x*^3^+*u*_*1*_*x*	*u*_*1*_ = −3*x*^2^	$X_{u_{1}}=u_{1}{}^{1/2}$
**Cusp model**	2	*V*(*x*) = *x*^4^+*u*_*1*_*x*^2^+*u*_2_*x*	*u*_*1*_ = −6*x*^2^,*u*_2_ = 8*x*^3^	$X_{u_{1}}=u_{1}{}^{1/2},X_{u_{2}}=u_{2}{}^{1/3}$
**Swallowtail model**	3	*V*(*x*) = 1/5*x*^5^+1/3*u*_*1*_*x*^3^+1/2*u*_2_*x*^2^+*u*_3_*x*	*u*_*1*_ = −6*x*^2^,*u*_2_ = 8*x*^3^,*u*_*3*_ = −3*x*^4^	$X_{u_{1}}=u_{1}{}^{1/2},X_{u_{2}}=u_{2}{}^{1/3},X_{u_{3}}=u_{3}{}^{1/4}$
**Butterfly model**	4	*V*(*x*) = 1/6*x*^6^+1/4*u*_1_*x*^4^+1/3*u*_2_*x*^3^+1/2*u*_3_*x*^2^+*u*_4_*x*	*u*_*1*_ = −10*x*^2^,*u*_2_ = 20*x*^3^,*u*_*3*_ = −15*x*^4^,*u*_4_ = 4*x*^5^	$X_{u_{1}}=u_{1}{}^{1/2},X_{u_{2}}=u_{2}{}^{1/3},X_{u_{3}}=u_{3}{}^{1/4},X_{u_{4}}=u_{4}{}^{1/5}$

Notes: Source from Woodstock and Poston (1974)

When performing recursive computations, the principle of minimum values or the principle of mean values is selected after determining if the indicators are complementary to each other, which means the impacts of different indicators in the same subsystem on the upper indicator can be replaced by each other or interchangeable within one subsystem. Formulas are used to perform recursive computation on the normalized data. In the end, conclusions are reached by comparison of different catastrophe fuzzy membership functions from a variety of judgment systems [24]. The total catastrophe fuzzy membership functions are a group of relative quantities with initial data that varies for the underlying layers but will not affect the final results.

### Development of a catastrophe model

#### Indices selection

The selection of indices was based on the comprehensive reference of Su et al [17], Wang et al [26], and Ni et al [27] focusing on contamination source, contamination levels and the relative effects on living things. Su et al [17] applied the ‘‘Pressure-State-Response” framework to assess land ecological security, guided by the principles of integrity, simplicity, dynamic response, geographical accuracy, and data availability. Wang et al [26] used catastrophe theory to determine fuzzy membership functions that define the relationship between state variable (water security) and control variables (climate change, economic development, population growth, etc.). Ni et al [27] chose chemical indices, physical indices, biological indices and water activity as the four control variables of water quality, the state variable, according to the environmental criteria and water quality monitoring requirements and so on.

Soil ecological security includes interactions between contamination source of the soil, the soil and living things in the soil. The source of the contamination could affect the contamination level of the soil, quality of vegetables planted in the plastic greenhouses by means of absorption of stomata on leaves and also the diversity and activity of soil animals, which is too large a group that we will not consider it in this study, and soil microorganisms. On the other hand, PAEs concentration levels in the soil could directly affect the concentration in vegetables by means of plant absorbing, the community status of soil organisms and even the atmospheric concentration of target contaminants, so a better understanding of the ecological security risk should be based on the understanding of all factors influence it.

**Fig 1:**
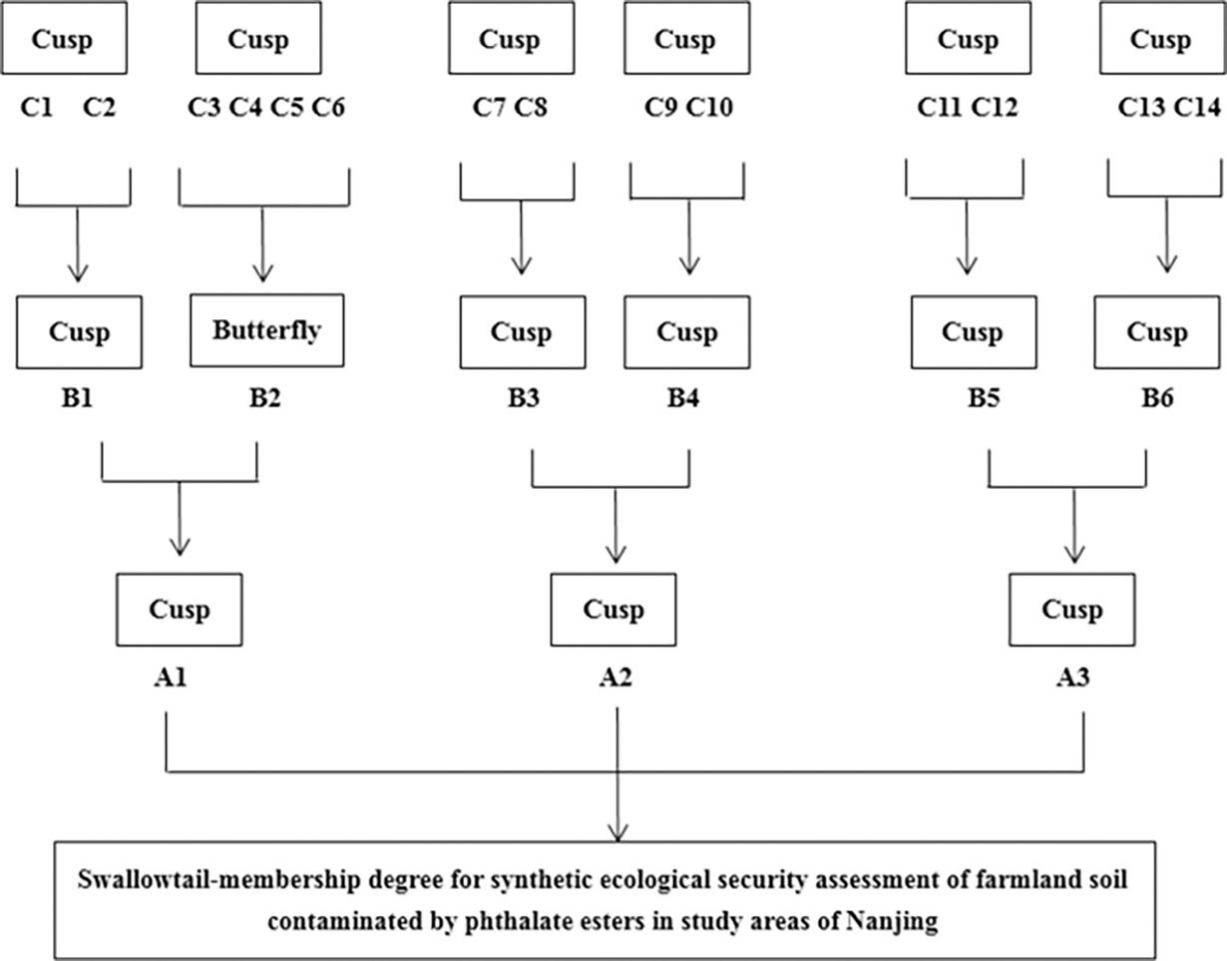
Catastrophe model for ecological security assessment of farmland soil contaminated by phthalate esters in study areas of Nanjing. A, B, and C denote different levels of indices in catastrophe model. Refer the details to “Indices selection” part and Table 2.

In the present study, it is considered that the contamination source, contamination status and the contamination effects often work mutually independent (Table 2), however, the interaction and restriction between them always exist within the system (Fig 1). Previous studies and their framework above have been referred to generate a set of assessment indices. After performing principle component analysis to reduce dimensional of the data, a total of 25 indices were generated (Tables 2, 3 and 4). Corresponding values between assessment results of catastrophe model and modified catastrophe model have been listed in Table 5. Accordingly, the assessment results of the soil ecological security could be divided into the following four levels: less than 0.80 (secure), 0.80 ~ 0.90 (middle secure), 0.90 ~ 0.95 (insecure) and higher than 0.95 (very insecure).

**Table 2:** Indicator system for ecology security assessment in the study area.

**No.**	**Element**	**No.**	**Items**	**No.**	**Indices**	**No.**	
**A1**	Contamination Source	B1	Inside	C1	Atmospheric Deposition	D1	Thickness
						D2	Melt Index
				C2	Irrigation	D3	Sewage Wastewater
						D4	Underground water
		B2	Outside	C3	Planting Mode	D5	Planting Age
						D6	Plastic film mode
				C4	Climate Condition	D7	Temperature
						D8	Rain Water
**A2**	Contamination Status	B3	In Soil	C5	Total Concentration	D9	PAEs
				C6	Single Concentration	D10	DEHP
						D11	DnBP
						D12	DnOP
		B4	In Vegetable	C7	Roots	D13	Turnip
						D14	Radish
				C8	Leafy	D15	Pakchoi
						D16	Chinese cabbage
						D17	Garlic bolt
						D18	Spinach
				C9	Solanaceous	D19	Cayenne
				C10	Stems	D20	Asparagus lettuce
**A3**	Contamination Effects	B5	Human	C11	Health Risk	D21	HQ values
		B6	Microorganisms	C12	Soil Microbial Counts	D22	Bacteria
						D23	Fungi
				C13	Microorganism Diversity Indices	D24	Shannon
						D25	McIntosh

Notes: The abbreviations are also used for other tables and figures.

**Table 3:** Statistical data used in ecological security assessment model of farmland soil contaminated by phthalate esters in study areas of Nanjing.

Indices	No.	Raw Data				Standardization Data				Normalization Data			
		GL	HS	PLK	SS	GL	HS	PLK	SS	GL	HS	PLK	SS
**Thickness of Plastic Films (mm)**	D1	0.011	0.009	0.016	0.016	0.71	1.00	0.00	0.00	0.85	1.00	0.00	0.00
**Melt Index of Plastic Films (g 10 min**^**-1**^**)**	D2	5.50	7.00	2.80	3.00	0.64	1.00	0.00	0.05	0.86	1.00	0.00	0.36
**Sewage Wastewater irrigate**	D3	0.50	0.50	0.00	0.00	0.50	0.50	0.00	0.00	0.71	0.71	0.00	0.00
**Underground Water irrigate**	D4	0.50	0.50	0.00	0.00	0.50	0.50	0.00	0.00	0.79	0.79	0.00	0.00
**Age (yr)**	D5	5.00	2.50	8.50	11.00	0.29	0.00	0.71	1.00	0.54	0.00	0.84	1.00
**Plastic Film Mode (year)**	D6	10.00	1.46	2.83	11.00	0.90	0.00	0.14	1.00	0.96	0.00	0.52	1.00
**Temperature (°C)**	D7	4.60	4.60	4.20	4.60	1.00	1.00	0.00	1.00	1.00	1.00	0.00	1.00
**Rain (mm)**	D8	16.00	16.00	17.00	16.00	0.00	0.00	1.00	0.00	0.00	0.00	1.00	0.00
**PAEs Total Concentration in Soil (mg kg**^**-1**^**)**	D9	2.07	2.56	1.29	0.94	0.70	1.00	0.22	0.94	0.70	1.00	0.22	0.94
**DEHP Concentration in Soil (mg kg**^**-1**^**)**	D10	0.98	0.91	0.39	0.25	0.98	0.91	0.39	0.25	0.99	0.95	0.62	0.50
**DnBP Concentration in Soil (mg kg**^**-1**^**)**	D11	0.46	0.69	0.42	0.33	0.46	0.69	0.42	0.33	0.77	0.88	0.75	0.69
**DnOP Concentration in Soil (mg kg**^**-1**^**)**	D12	0.01	0.01	0.01	0.01	0.01	0.01	0.01	0.01	0.32	0.32	0.32	0.32
**PAEs Total Concentration in Turnip (mg kg**^**-1**^**)**	D13	1.47	1.25	0.82	1.46	1.00	0.66	0.82	0.98	1.00	0.82	0.91	0.99
**PAEs Total Concentration in Radish (mg kg**^**-1**^**)**	D14	1.54	2.55	0.52	1.54	0.50	1.00	0.52	0.50	0.80	1.00	0.80	0.80
**PAEs Total Concentration in Pakchoi (mg kg**^**-1**^**)**	D15	2.72	1.34	0.73	1.60	1.00	0.31	0.73	0.44	1.00	0.55	0.85	0.66
**PAEs Total Concentration in Chinese cabbage (mg kg**^**-1**^**)**	D16	1.73	1.56	1.65	1.65	1.00	0.00	0.53	0.53	1.00	0.00	0.81	0.81
**PAEs Total Concentration in Garlic bolt (mg kg**^**-1**^**)**	D17	3.07	2.28	1.48	2.28	1.00	0.50	0.00	0.50	1.00	0.84	0.00	0.84
**PAEs Total Concentration in Spinach (mg kg**^**-1**^**)**	D18	2.13	1.42	0.82	1.31	1.00	0.46	0.82	0.37	1.00	0.86	0.96	0.82
**PAEs Total Concentration in Cayenne (mg kg**^**-1**^**)**	D19	0.81	2.73	1.44	0.78	0.81	1.00	0.34	0.78	0.81	1.00	0.34	0.78
**PAEs Total Concentration in Asparagus lettuce (mg kg**^**-1**^**)**	D20	4.04	2.93	0.69	1.28	1.00	0.67	0.69	0.18	1.00	0.67	0.69	0.18
**HQ values**	D21	4.53	4.79	1.74	1.35	0.92	1.00	0.11	0.00	0.92	1.00	0.11	0.00
**Bacteria Count (×10**^**7**^**)**	D22	2.05	3.61	2.63	2.88	0.00	1.00	0.37	0.53	0.00	1.00	0.61	0.73
**Fungi Count (×10**^**6**^**)**	D23	4.25	3.97	4.02	4.57	0.47	0.00	0.08	1.00	0.78	0.00	0.44	1.00
**Shannon Index**	D24	2.09	2.85	2.41	2.48	0.00	1.00	0.42	0.51	0.00	1.00	0.65	0.72
**McIntosh Index**	D25	1.50	1.72	1.58	1.62	0.00	1.00	0.36	0.55	0.00	1.00	0.71	0.82

Notes: Two digital valid numbers have been left in the standardization table but kept intact during the calculation. Meteorological data are from https://15tianqi.cn/2011jiangning12yuetianqi/; https://15tianqi.cn/2011nanjinglishui12yuetianqi/. Refer the abbreviations to Table 2.

**Table 4:** Calculated data of indicators of the setup catastrophe model.

No.	GL	HS	PLK	SS	No.	GL	HS	PLK	SS	No.	GL	HS	PLK	SS
**C1**	0.85	1	0	0.18	B1	0.92	0.95	0	0.21	A1	0.95	0.86	0.47	0.71
**C2**	0.75	0.75	0	0										
**C3**	0.75	0.00	0.68	1.00	B2	0.83	0.40	0.81	0.90					
**C4**	0.50	0.50	0.50	0.50										
**C5**	0.70	1.00	0.22	0.94	B3	0.42	0.50	0.23	0.48	A2	0.82	0.84	0.72	0.83
**C6**	0.70	0.72	0.56	0.50										
**C7**	0.90	0.91	0.85	0.89	B4	0.97	0.93	0.87	0.88					
**C8**	1.00	0.56	0.66	0.78										
**C9**	0.81	1.00	0.34	0.78										
**C10**	1.00	0.67	0.69	0.18										
**C11**	0.92	1.00	0.11	0.00	B5	0.92	1.00	0.11	0.00	A3	0.82	0.97	0.63	0.49
**C12**	0.39	0.50	0.52	0.86	B6	0.31	0.85	0.80	0.92					
**C13**	0.00	1.00	0.68	0.77										

Notes: Refer the abbreviations to Table 2.

**Table 5:** Corresponding values between assessment results of catastrophe model and modified catastrophe model.

Health risk level	Relative degree obtained by catastrophe model and modified catastrophe model
**Very insecure**	>0.95
**Insecure**	0.90 ~ 0.95
**Middle**	0.80 ~ 0.90
**Secure**	<0.80

#### Data source

Statistical data in December 2011 obtained in our laboratory have been used in this paper [6,28]. In the collection of all data, main principles are as follows.

1. Contamination source

Both the thickness (D1) and the melt index (D2) of the plastic films are important parameters to evaluate the quality and in accordance with PAEs release to the soil from atmospheric deposition, proved by Ma et al before [6]. In planting mode, planting age (D3) is an average time of greenhouse business operation, and the data of plastic film mode were from Wang et al [28]. In GL, two layers of plastic films covered the greenhouse in the whole year, so in 5 years of planting, the value is calculated as 10; in HS, one lay of plastic film was used only in 7 months of the year (7/12), so in the 2.5 years of planting, the value is the product of them, 1.46; in PLK, one layer of plastic film was used every two years, and discarded in the third year, which is equivalent to 1/3 in the production of 8.5 years, so the value is 2.83; in SS, concerning about the planting years 11 and one layer of plastic films all over the year, the value of plastic film mode is 11. Temperature and rain generally reference to the local information of the sampling month.

1. Contamination status

In B3 (contamination status in soil), although the total contamination level of PAEs in soil is very important in the assessment of ecological security risk, the single concentration of the large proportion composition also plays a crucial role, and the toxicity of every PAE compound represent different weights. The sequence of contamination level of vegetables depends mainly on the consumption ratio and quantity from C7 (contamination status in vegetable, roots) to C10 (contamination status in vegetable, stems), however, for example, D13 (contamination status in vegetable, roots, turnip) to D16 (contamination status in vegetable, leafy, Chinese cabbage), the weights was decided by the comprehensive planting ratio of different vegetables in the four selected areas.

1. Contamination effects

Except the inestimable soil animal group, the effects of target pollutants to human being and soil organisms are supposed to be much easier obtained and reliable. Microbial counts and diversity are the intuitive form of expression of soil organism community in contaminated soil [29].

Dilution plate counting method were conducted in the counting of bacteria and fungi. After the soil samples were passed through a sieve of 1.7 mm mesh, the bacteria were originally isolated by plating dilutions in saline solution (0.9% NaCl) on nutrient agar containing glucose, 10 g L^-1^, NaCl, 1 g L^-1^, peptone, 5 g L^-1^, yeast extract, g L^-1^, and agar 20 g L^-1^, before incubated at 37°C for 48 h. The developed colonies were counted in plates and the average number of colonies per three plates was determined. The number of total bacteria (CFU) per gram dry weight of soil was determined [30].

For fungal count determination, 10 g soil was put into 90 mL sterile water, stirred vigorously for 30 min, diluted serially, and plated on the Martin medium containing Rose Bengal and 2 mL L^-1^ lactic acid. After incubated at 25°C for 4 d, the colony number of soil fungal populations on the plates was counted. All the results were the mean values of three replicate determinations and expressed on an oven-dry weight basis [31].

Biolog Eco plates were used to study the substrate utilization pattern of soil microbial communities. Fresh soil (5 g) from each was sieved and added to 100 mL of distilled water in a 250 mL conical flask and shaken at 200 rev min^-1^ for 20 min. Tenfold serial dilutions were made, and1000-fold soil dilution solution was used for injection into the wells of the Biolog Eco plates. Plates were incubated at 25°C for 7 days, and color development was measured daily as difference in absorbance (A) at 590 nm using a μQuant microplate spectrophotometer (BioTek, Winooski, VT) before the data were calculated using Gen5v1.06 software [32].

The Shannon index reflects both the substrate richness and the substrate evenness of test microorganisms. Shannon measures are shown to be the only standard diversity measures that can be decomposed into meaningful independent alpha and beta components when community weights are unequal [33]. Shannon diversity index:

$$H=-\sum_{i}^{n}P_{i}\times lnP_{i}$$

Pi—each reaction well subtracting the absorbance value of the control well and then dividing by the summed color absorbance value of 31 wells.

McIntosh index [34]:

$$U=\sqrt{\left(\sum_{i=1}^{n}p_{i}^{2}\right)}$$

#### Data standardization

Since the range and units of original data are different from each other, the value of index is converted to a dimensionless number ranges from 0 to 1 by raw data standardization. Normalization is done by using different formulas for different types of indicators. For the indicators which indicate better condition when their values are large, a formula known as “larger is better” (ILB) is used, which is as follows:

$$Y=\{\begin{array}{l} 1\;0\leq X\leq a_{1}\\ \left(a_{2}-X)/(a_{2}-a_{1}\right)\;a_{1}\leq X\leq a_{2}\\ 0\;X\geq a_{2} \end{array}$$ (1)

For the indicators which indicate better condition when their values are smaller, a formula known as “smaller is better” (ISB) is used, which is as follows:

$$Y=\left\{ \begin{array}{l} 1\;X\geq a_{2}\\ (X-a_{1})/(a_{2}-a_{1})\;a_{1}<X<a_{2}\\ 0\;0\leq X\leq a_{1} \end{array}\right.$$ (2)

For the indicators which cannot be categorized according to above two groups, a formula known as “All is ok” (IOB) is used, which is as follows:

$$Y=\left\{ \begin{array}{l} 2(X‑a_{1})/(a_{2}‑a_{1})\;a_{1}\leq X\leq a_{1}+(a_{2}-a_{1})/2\\ 2(a_{2}‑X)/(a_{2}‑a_{1})\;a_{1}+(a_{2}-a_{1})/2<X\leq a_{2}\\ 0\;X>a_{2}\;or\;X<a_{1} \end{array}\right.$$ (3)

where, a1 and a2 are the upper and lower bounds of the function. In practical evaluation, 10% lower than the maximum value of a quantitative indicator is considered as its upper bound and 10% higher than the minimum value of a quantitative indicator is considered as its lower bound.

#### Modification of catastrophe model

In some circumstances, the indices are not so sufficient provided, so that the original catastrophe model should be modified to a simpler one for wider applicability (Tables 6 and 7). Under the condition without contamination effects data and pollution status in plants (vegetables), indices D15 to D17 could be deleted. D13 could be transformed to DEHP concentration in plants (mg kg^-1^) and D14 could be transformed to DnBP concentration in plants (mg kg^-1^) (Table 6). And the final evaluation data of the year 2012 in four study areas under the modified model version have been listed in Table 7 to test the reliable of the model.

**Table 6:** Application of the ecological security assessment model on the same farmland soil based on the data of 2012.

Indices	No.	Raw Data				Standardization Data				Normalization Data			
		GL	HS	PLK	SS	GL	HS	PLK	SS	GL	HS	PLK	SS
**Thickness of Plastic Films (mm)**	D1	0.011	0.009	0.016	0.016	0.71	1.00	0.00	0.00	0.85	1	0	0
**Melt Index of Plastic Films (g 10 min**^**-1**^**)**	D2	5.50	7.00	2.80	3.00	0.64	1	0	0.05	0.86	1.00	0	0.36
**Sewage Wastewater irrigate proportion**	D3	0.50	0.50	0	0	0.5	0.5	0	0	0.71	0.71	0	0
**Underground Water irrigate proportion**	D4	0.50	0.50	0	0	0.5	0.5	0	0	0.79	0.79	0	0
**Age (yr)**	D5	5.00	2.50	8.50	11.00	0.29	0	0.71	1	0.54	0	0.84	1.00
**Plastic Film Mode (year)**	D6	10.00	1.46	2.83	11.00	0.90	0	0.14	1	0.96	0.00	0.52	1.00
**Temperature (°C)**	D7	2.90	2.90	3.20	2.90	0.00	0.00	1.00	0.00	0.00	0.00	1.00	0.00
**Rain (mm)**	D8	100.00	100.00	97.00	100.00	1.00	1.00	0.00	1.00	1.00	1.00	0.00	1.00
**PAEs Total Concentration in Soil (mg kg**^**-1**^**)**	D9	3.18	0.81	3.16	1.87	1.00	0.81	0.99	0.45	1.00	0.81	0.99	0.45
**DEHP Concentration in Soil (mg kg**^**-1**^**)**	D10	2.09	0.54	3.04	1.02	0.62	0.54	1	0.192	0.79	0.73	1.00	0.44
**DnBP Concentration in Soil (mg kg**^**-1**^**)**	D11	0.14	0.07	0.13	0.15	0.14	0.07	0.13	0.15	0.52	0.41	0.51	0.53
**DnOP Concentration in Soil (mg kg**^**-1**^**)**	D12	0.34	0.05	0.00	0.04	0.34	0.05	0	0.04	0.76	0.47	0.00	0.45
**DEHP Concentration in plants (mg kg**^**-1**^**)**	D13	0.16	0.62	0.13	0.27	0.16	0.62	0.13	0.27	0.40	0.79	0.36	0.52
**DnBP Concentration in plants (mg kg**^**-1**^**)**	D14	0.05	0.12	0.06	0.07	0.05	0.12	0.06	0.07	0.37	0.49	0.39	0.41

Note: https://15tianqi.cn/2012jiangning1yuetianqi/; https://15tianqi.cn/2012nanjinglishui1yuetianqi/. Refer the abbreviations to Table 2.

**Table 7:** Calculated data of indicators of the setup catastrophe model.

No.	GL	HS	PLK	SS	No.	GL	HS	PLK	SS	No.	GL	HS	PLK	SS
**C1**	0.85	1	0	0.18	B1	0.92	0.95	0	0.21	A1	0.95	0.86	0.47	0.71
**C2**	0.75	0.75	0	0										
**C3**	0.75	0	0.68	1	B2	0.83	0.40	0.81	0.90					
**C4**	0.5	0.5	0.5	0.5										
**C5**	1.00	0.81	0.99	0.45	B3	0.50	0.45	0.50	0.33	A2	0.72	0.77	0.71	0.68
**C6**	0.69	0.54	0.50	0.47										
**C7**	0.38	0.64	0.38	0.47	B4	0.38	0.64	0.38	0.47					

Note: Refer the abbreviations to Table 2.

### Application of catastrophe theory

Catastrophe theory is no longer new but it remains controversial. Controversy arises not out of the mathematical foundations of the theory nor from applications in the hard sciences, but principally when it is applied to the social and biological sciences [35]. Understanding discontinuities helps to better comprehend biological systems, and catastrophe theory deals with discontinuities and provides a way of modelling them [36]. It is, therefore, found that the catastrophe has both properties of phase transition and indirect effect [37].

### Statistical analysis

All data were processed with Microsoft Excel 2016 and the SPSS v.18.0 software package. Pairs of mean values were compared for significant differences using the least significant difference (LSD) method at the 5% level.

## Results and discussion

### Contamination status of PAEs in soils and vegetables

**Fig 2:**
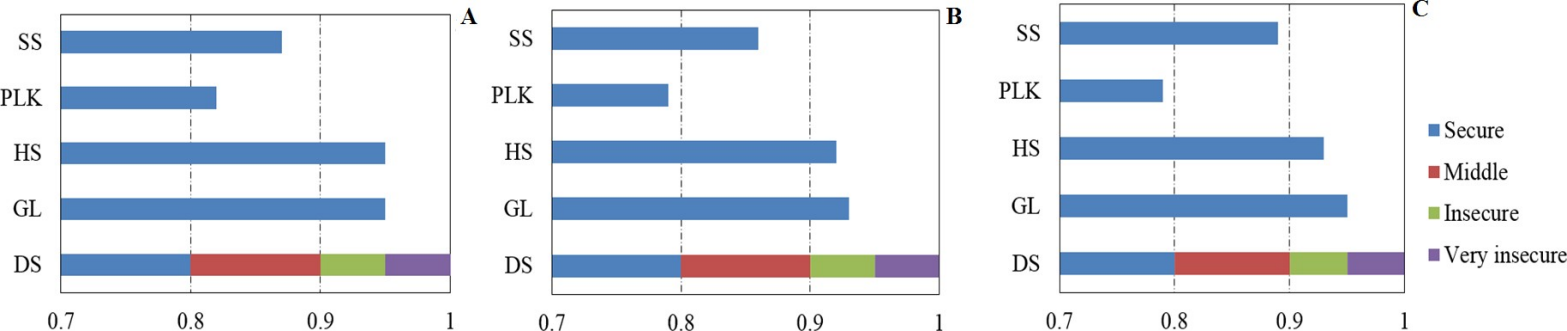
Calculated results of relative degree obtained by catastrophe model and modified catastrophe model in four study areas. The abbreviations are Gu Li village (GL), Hu Shu village (HS), Planck farm (PLK), Suo Shi village (SS) and dividing standard (DS). (A) final calculated data of the year 2011 under the setup catastrophe model; (B) final calculated data of the year 2012 under the modified catastrophe model; and (C) final data of the year 2012 under the modified model.

In Tables 3 and 4 and Fig 2A, the initial data collected in December, 2011 [6] and the annual statistical meteorological data of the indicators in the setup catastrophe model describing A1) contamination source, A2) contamination status and A3) contamination effects of the soils in four study areas in suburb Nanjing were listed. Tables 6 and 7 and Fig 2B and 2C showed the initial data collected in January, 2012 [28,38].

The concentration data of soil and vegetable of the four typical protected agriculture areas in suburb Nanjing, reveal the serious level of PAE contamination [6], which are the main contribution of soil ecology risk in study areas. As for soils, the PAE concentration exhibits a higher relationship with film quality, continuous planting, and greenhouse management mode [6]. As for vegetables, PAEs accumulation depends mainly on the physical and chemical properties of these compounds in addition to the factors such as adsorption pathways, soil PAEs concentration, soil properties, growing period, plant concentrate capabilities, and so on [6]. Root vegetables accumulated some PAE compounds by mainly direct contact with the contaminated soil [6]. Eating raw vegetables, without the elimination of PAEs by cooking or heating, might increase the PAE risk to human health [6]. Based on the results of the investigation and risk assessment, DEHP presents the highest health risk, and risk caused by soil ingestion is relatively high and more difficult to avoid [6], followed by DnBP. According to the conclusions of the previous study, the selection and setting of the parameters in catastrophe model could be reasoned.

### Computation results from (modified) catastrophe model

According to the evaluation index data in Fig 1, the catastrophe models used for each index are as follows. 1) D1 (ISB), D2 (ILB) and C1 make up a cusp model, and D1 and D2 are non-complementary; 2) D3 (ILB), D4 (ILB) and C2 make up a cusp model, and D3 and D4 are complementary; 3) D5 (ILB), D6 (ILB) and C3 make up a cusp model, and D5 and D6 are non-complementary; 4) D7 (ILB), D8 (ILB) and C4 make up a cusp model, and D7 and D8 are complementary; 5) D9 (ILB) and C5 make up a fold model; 6) D10 (ILB), D11 (ILB), D12 (ILB) and C6 make up a swallowtail model, and D10, D 11 and D12 are complementary; 7) D13 (ILB), D14 (ILB) and C7 make up a cusp model, and D13 and D14 are complementary; 8) D15 (ILB), D16 (ILB), D17 (ILB), D18 (ILB) and C8 make up a butterfly model, and D15, D16, D17 and D18 are complementary; 9) D19 (ILB) and C9 make up a fold model; 10) D20 (ILB) and C10 make up a fold model; 11) D21 (ILB) and C11 make up a fold model; 12) D22 (ISB), D23 (ILB) and C12 make up a cusp model, and D22 and D23 are complementary; 13) D24 (ISB), D25 (ISB) and C13 make up a cusp model, and D24 and D25 are complementary.

In Table 3, raw data of D3 in GL, HS, PLK and SS were 0.50, 0.50, 0 and 0, indicating that the sewage wastewater irrigation ratio is half for GL and HS, none for PLK and SS. And the raw data of D4 in the same table were 0.50, 0.50, 0 and 0, indicating that the underground water irrigate is half for GL and HS, entirely for PLK and SS.

### Analysis of the computation results

As shown in Tables 3 and 4 and Fig 2A, the final ecological security factors of the four study areas GL, HS, PLK and SS were 0.95, 0.95, 0.82 and 0.87. According to the reference values in Table 5, the soil environment ecology security degree of GL, HS, PLK and SS in December 2011 were insecure, insecure, middle and middle, respectively. Among all the referred data, thickness and melt index of plastic films are the most crucial parameters. Thicker plastic films with lower melt index, which means less quantity of plastic films could be melt within 10 min, indicating the relative stability of the films, could significantly decrease the final assessment result. Thicker plastic films with lower melt index could significantly decrease the final assessment result, suggesting the application of plastic films with better quality could largely save soil ecology from been ruined.

On the other hand, the contamination of PAEs in irrigation water was another reason for the increasing of introduction rate and contamination risk of target pollutants. The computation results objectively reflect the practical production and operating situation of the four study areas in 2011, and the conclusion based on the questionnaire during the investigation have also proved the reliability of the assessment because the operating condition and growth condition of vegetables in GL and HS is obviously worse than that of PLK, which has the least value and the highest security.

As shown in Tables 6 and 7 and Fig 2B and 2C, the final ecological security factors of the four study areas GL, HS, PLK and SS calculated based on the modified catastrophe model and the initial catastrophe model have been listed and compared. Under modified catastrophe model the assessment results of the raw data obtained in January 2012 were 0.93, 0.92, 0.79 and 0.86 (Fig 2B), and under the initial catastrophe model, the results were 0.95, 0.93, 0.79 and 0.89 (Fig 2C). Firstly, the interval of determine the two groups of raw data was about a month, so that the assessment results would not change much. In this study, compared with the 0.95, 0.95, 0.82 and 0.87 also obtained under the initial catastrophe model using data of December 2011 (Fig 2A) [6], the security degree results were almost at the same level in the four study areas GL, HS, PLK and SS based on the data of January 2012 [25]. Secondly, the calculated results using the initial and modified model were also at the same level in the four study areas based on the same raw data source of January 2012. When the raw data of the different sampling time were compared, we could see the variations from D7 to D14. However, no significant differences have been found after the calculation no matter using different models or data from different time (*p*<0.05). This computation result objectively reflects the security degree of PAEs in soil ecology in study areas, and the soil ecology security situation in GL and HS is insecure from December 2011 on.

In the previous study, the comparison of hazard quotient (HQ) values reveals the results of risk assessment of six PAEs in four study areas. Calculated HQ of the total PAEs in the four investigated areas indicated that DEHP showed the highest potential health risk to local children aged between 0 and 6, followed by DnBP in HS [6]. Total risk of PAE exposure was in the order of GL>HS>SS>PLK in the four investigated areas, which is in good correlation with results achieved from catastrophe model from the four selected areas in Nanjing, no matter under the initial catastrophe model or the modified one. The comparison result could indirectly prove better accuracy and reliability of this model, because the standard comparison system in this study with several division values is definitely more precise than the HQ assessment system by comparing with one threshold value.

## Conclusions

In the initial catastrophe model we developed, the subjectivity of the computation in soil ecology security has been decreased. In the application of the setup models in this study on assessment of data achieved in January 2012, the results were 0.95, 0.93, 0.79 and 0.89 under the initial catastrophe model, and 0.93, 0.92, 0.79 and 0.86 under modified catastrophe model in GL, HS, PLK and SS, which is consistent with the results of previous studies. The results confirmed the reliability of catastrophe theory to the field of soil environmental ecological security assessment. However, in order to achieve more accurate and precise assessment results, collecting of more detail data and information in order to apply the initial setup model is more recommended. Based on the cause and effect contamination raw data from other study areas, the general soil ecological security degree could be obtained use methods described herein. Thanks to the assessment results of the setup catastrophe model for PAE pollutants, guiding policy on plastic film selection, agricultural food security and human health risk could be roughly understood according to the priority of selected indicators.

## Supporting information

S1 File Highlights. (DOCX)

S2 File 1-normalization-origin model. (XLSX)

S3 File 2-normalization-modified model. (XLSX)
