# Supplementary material for: Application of catastrophe theory in comprehensive ecological security assessment of plastic greenhouse soil contaminated by phthalate esters
Source: PLoS One. 2018 Oct 31;13(10):e0205680. doi: 10.1371/journal.pone.0205680 (PMC6209207; doi:10.1371/journal.pone.0205680)
Supplement: S1 File — (DOCX) [file pone.0205680.s001.docx]

**Highlights**

PAEs in films of facility agriculture cause damage to different organisms.

A new frame work has been put forward to measure soil ecology security risk.

An evaluation system was composed of thirteen mesosphere indicators.

A modified evaluation system was improved based on less data need.

The results proved the reliability in practical study.
